# Intralesional injection of hyaluronic acid compared with verapamil in acute phase of Peyronie’s disease: A prospective randomized clinical trial

**DOI:** 10.1080/20905998.2024.2333583

**Published:** 2024-03-22

**Authors:** Ahmed Abou Elezz Abdel Fattah, Tamer Diab, Amr S. El-Dakhakhny, Salah A. El Hamshary

**Affiliations:** Urology Department, Faculty of Medicine, Benha University, Benha, Egypt

**Keywords:** Intralesional injection, hyaluronic acid, verapamil, acute phase, Peyronie’s disease

## Abstract

**Purpose:**

The aim of this work was to analyze and contrast the effectiveness and safety of intralesional HA in the acute stage of PD with that of verapamil injection.

**Methods:**

In this prospective, randomized clinical trial, 42 PD-affected, sexually active men between the ages of >18 and 70 participated. Two groups of patients were recruited; group A obtained weekly intralesional treatment with HA for 12 weeks, whereas group B obtained weekly intralesional therapy with verapamil for 12 weeks. Physical examinations and Duplex Doppler ultrasound were performed on all patients.

**Results:**

The penile curvature was significantly decreased at 12 weeks after therapy in contrast to baseline in group A (34.1 ± 6.77° vs. 24.7 ± 9.72°, *p* = 0.005), and was significantly decreased at 12 weeks after therapy compared to baseline in group B (36.2 ± 7.43° vs. 30.8 ± 8.63 °, *p* = 0.047). The decrease in penile curvature at 12 weeks after therapy was noticeably better in group A in contrast to group B (24.7 ± 9.72° vs. 30.8 ± 8.63°, *p* = 0.038).

**Conclusion:**

HA is emerging as a valid choice for the treatment of PD in terms of resolution of the acute phase of the disease, and it is plausible to posit that the use of HA may contribute to the stabilization of the disease and decrease the need for the subsequent choice of a possible surgical strategy, with the ability to reduce penile pain and have a stronger impact on penile curvature and patient satisfaction.

## Introduction

The term Peyronie’s disease (PD) refers to a long-lasting benign disease that causes localised fibrous inelastic scars to develop to the penis’ tunica albuginea level. This disorder may result in erectile dysfunction (ED), penile curvature, and painful erections [1]. PD is thought to impact between 3 and 9% of males, and it is more common in people with cardiovascular disease and diabetes [2].

It is not understood what causes PD. Current popular theories suggest that plaque formation is the result of a low-level autoimmune response resulting from a lengthy, intricate inflammatory response of the tunica albuginea fibres to a single traumatic event or to numerous minor injuries experienced throughout a sexual act [3]. The PD has two states, an acute phase and a steady state. The acute phase, which can continue for up to 18 months, is when plaques form. In the chronic stage, a decline in penile pain is expected with a stabilization of penile deformity. When a patient’s curvature has been stable for at least three months, they have entered the chronic phase [4].

Both medicinal and surgical methods are used to treat PD, with the method used depending on the stage of the disorder, the severity of the deformity, and other factors [5,6]. Patients in the early phase of PD benefit most from conservative treatment, and those with stable illness for at least 12 months might consider surgical remediation to rectify curvature and improve their capacity for fulfilling sexual interaction [7].

The most popular treatments for the acute stage of PD are intralesional injections of corticosteroids and verapamil [8,9]. The most effective treatment for penile plaques is injecting pharmacologically active drugs directly into the plaques themselves. Guidelines from the American Association of Urology recommend using Clostridium histolyticum collagenase (CHC) intralesionally in conjunction with modelling or intralesional interferon alfa-2b or verapamil [10]. Penile ecchymosis, swelling, pain, and corporal rupture with collagenase; sinusitis, dizziness, minor penile swelling with interferon alfa-2b; pain at the injection site, penile bruising, and nausea with verapamil have all been reported as possible side effects of these compounds. The expensive price of collagenase further restricts its use in therapy [11,12].

Hyaluronic acid (HA) appears to be helpful at inhibiting the inflammatory and oxidative stress mediators’ detrimental effects on scar formation. In particular, oxidative stress appears to be essential for the development of PD, inducing upregulation of fibrogenic cytokines and increasing collagen synthesis in the early stage of PD [13,14].

Intralesional injections of verapamil were introduced by Levine et al. [15], this calcium antagonist, reducing calcium-dependent collagen transport and collagenase activities, seems to have the capacity to slow, prevent, or even reverse plaque formation and the progression of PD. Since then, studies have assessed the efficacy of ILVI for PD but have yielded mixed results. While initial reports showed significant improvements in curvature, later studies failed to display similar significant outcomes [16].

To the best of my knowledge, our study is an evolving management of PD in its early stages rather than waiting till the establishment of fibrosis, hence the patients may need surgical correction and may also need application of penile prosthesis.

This study’s objective was to establish if acute intralesional injections of HA could slow the progress of PD by inhibiting inflammatory and pro-fibrotic processes. Because of this, we decided to carry out this research to contrast the efficiency and safety of intralesional HA to that of verapamil injection in individuals experiencing the acute stage of PD.

## Patients and methods

Forty-two sexually active men aged 18 and up with PD were enrolled in this prospective, longitudinal, double-blind, randomised clinical research. Patients gave their written approval before participation (Approval code: RC 6-5-2023).

Patients were incorporated if they were older than 18 years, had a progressive penile curvature of more than 15 degrees in the last 12 months, had pain in the flaccid state or during painful erections, and had a palpable nodule or plaque in the tunica of penis. Exclusion criteria included patient refusal, calcified plaques or hourglass deformity as determined by duplex Doppler ultrasonography, prior PD therapy with intralesional injections, congenital penile curvature, history of prior penile surgery, and concurrent oral treatment for PD.

## Randomization

A computer-generated sequence was employed to divide the patients into two equal groups and seal opaque envelopes: According to the previous line of treatment, patients were randomly split into two groups. Group A underwent a once-weekly therapy for 12 weeks with intralesional HA (0.8% extremely pure sodium salt HA 16 mg/2 mL; Sinovial, IBSA, Lodi, Italy). Verapamil (10 mg in 5 mL of ordinary saline water) was administered intralesionally to group B once every week for a total of 12 weeks. Participants and researchers were both blinded.

A thorough history was taken for every patient (age, BMI), history of comorbidities (HTN, diabetes, and dyslipidaemia), symptoms as chest pain and dyspnea and previous medication. Clinical examination and laboratory investigation were assessed. Initial eligibility assessment, which includes medical, sexual histories, and physical examination.

## Patients’ evaluation

Duplex Doppler ultrasonography was performed on all patients both before and after receiving an intracorpus injection of a tri-mix (papaverine 30 mg/mL, phentolamine 1 mg/mL, and alprostadil 10 mg/mL) to cause penile erections. It took a maximum of three doses of repeat dosing to acquire complete erectile stiffness. At each centre, the same skilled operator carried out every procedure.

The longest diameter of the plaque was used to determine its size (in millimetres), and its position (proximal, medial, distal, or septum) was determined. A goniometer protractor was employed to gauge how curved the penile was at its stiffest. Acute inflammatory stage of the disease, a soft penile nodule or plaque, a painful erection, and/or a current alteration in penile curvature, was identified in each case. Each centre only had one urologist with experience doing all clinical and technical examinations. Dorsal, right-dorsal, left-dorsal, left-lateral, right-lateral, and ventral were all terms used to denote the bending side. A goniometer was employed to calculate the stiffest point’s curvature of the penile.

From the beginning of treatment through the end, alterations in plaque size, penile curvature and pain were evaluated. All of the subjects finished the treatment cycle and showed up for the three-month follow-up appointment to assess the effectiveness of the therapy.

## Main outcomes

The primary effectiveness result (degree) was a difference in penile curvature between baseline and endpoint (12 weeks after therapy). The change in plaque size (mm) was the secondary outcome.

## Sample size calculation

G. power 3.1.9.2 (Universität Kiel, Germany) was employed to calculate the sample size. According to a previous study, the sample size was determined by the changes in penile curvature from the baseline to the endpoint (our primary outcome), which had diminished of 4.60° (SD ± 5.63) from the baseline in the Hyaluronic acid group compared to no change in the Verapamil group (mean change was 4.60 ± 5.63 vs 0.00 ± 0.00, *p* < 0.001) [16]. 8 more cases were added to the allocation ratio of 1:1 based on 0.05 error and an 80% power of the trial to prevent dropout. Forty-two patients were allocated as a result, with 21 cases in each group.

## Statistical analysis

The SPSS v26 statistical analysis programme was used (IBM Inc., Armonk, NY, USA). Quantitative data from the two groups were compared utilizing unpaired Student’s t-test. Mean and standard deviation (SD) were the units of measurement for the quantitative variables. Chi-square test or Fisher’s exact test was utilized to evaluate qualitative data, which were provided as frequency and percentage (%), if applicable. When the two samples are correlated, a statistical technique utilized to contrast the two population means is the paired sample t-test. Statistical significance was set to 0.05 for two-tailed *p* values.

## Results

**Figure 1.**
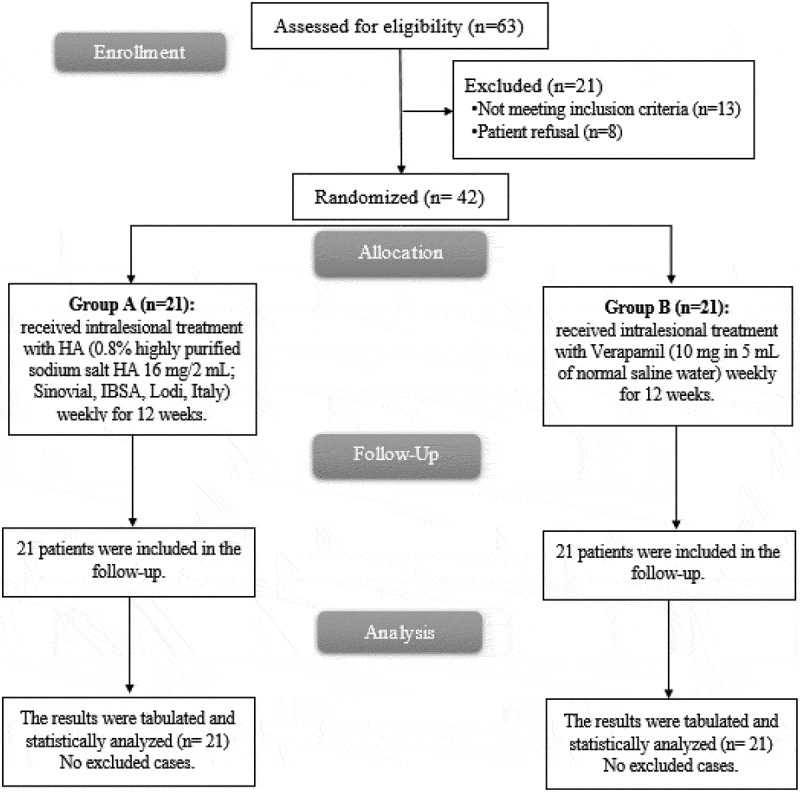
CONSORT flowchart of the enrolled patients.

In this study, 63 patients were evaluated for eligibility; 13 patients failed to fulfil the requirements, and 8 patients declined to take part in the trial. The other 42 patients were divided into 2 groups at random (21 patients in each). All assigned patients underwent observation and statistical evaluation. Figure 1.

Age, BMI, disease duration, and concomitant conditions (DM, cardiovascular disease) did not significantly differ across the study groups. Table 1.

**Table 1.** Baseline characterestics of the studied groups.

	Group A (*n* = 21)	Group B (*n* = 21)	P value
Age (years)	51.3 ± 5.94	52.8 ± 7.23	0.488
BMI (Kg/m^2^)	24.7 ± 1.68	23.95 ± 2.04	0.193
Duration of the disease	3.2 ± 1.1	2.7 ± 1.02	0.138
DM	13 (61.9%)	11 (52.38%)	0.755
Cardiovascular disease	7 (33.33%)	6 (28.57%)	0.738

Data displayed as mean ± SD or frequency (%), BMI: body mass index, DM: diabetes mellitus.

Regarding the clinical data, plaque position and curvature side were insignificantly different between the groups under study. Table 2.

**Table 2.** Clinical data of the studied groups.

		Group A (*n* = 21)	Group B (*n* = 21)	P value
Plaque position	Proximal	6 (28.57%)	5 (23.81%)	0.289
	Medium	2 (9.52%)	6 (28.57%)	
	Distal	13 (61.9%)	10 (47.62%)	
Curvature side	Dorsal	11 (52.38%)	14 (66.67%)	0.560
	Right lateral	5 (23.81%)	3 (14.29%)	
	Left lateral	2 (9.52%)	3 (14.29%)	
	Right lateral-dorsal	3 (14.29%)	1 (4.76%)	

Data presented as frequency (%).

At 12 weeks, the plaque size had considerably decreased after therapy compared to baseline in group A (7.7 ± 2.33 vs. 10.2 ± 2.32, *p* < 0.001) and was insignificantly different between 12 weeks after therapy and baseline in group B. The plaque size was significantly less in group A in contrast to group B at 12 weeks after therapy (7.7 ± 2.33 vs. 9.3 ± 2.37, *p* = 0.031).

The penile curvature was significantly decreased at 12 weeks after therapy in contrast to baseline in group A (34.1 ± 6.77° vs. 24.7 ± 9.72°, *p* = 0.005), and was significantly decreased at 12 weeks after therapy in contrast to baseline in group B (36.2 ± 7.43° vs. 30.8 ± 8.63°, *p* = 0.047). The decrease in penile curvature at 12 weeks after therapy was significantly better in group A in contrast to group B (24.7 ± 9.72° vs. 30.8 ± 8.63°, *p* = 0.038). At baseline, there was no statistically significant difference in plaque size or penile curvature between the two groups. Table 3.

**Table 3.** Plaque size and penile curvature of the studied groups.

		Group A (*n* = 21)	Group B (*n* = 21)	P value
Plaque size (mm)	Baseline	10.2 ± 2.32	9.9 ± 2.14	0.631
	12 weeks after therapy	7.7 ± 2.33	9.3 ± 2.37	0.031*
	P value within group	<0.001*	0.234	—
Penile curvature	Baseline	34.1 ± 6.77	36.2 ± 7.43	0.335
	12 weeks after therapy	24.7 ± 9.72	30.8 ± 8.63	0.038*
	P value within group	0.005*	0.047*	—

Data displayed as mean ± SD, *: statistically significant as *p* value < 0.05.

## Discussion

The best way to handle the acute period of PD is still up for debate. Despite the fact that surgery is still the preferred course of treatment for patients with stable PD, it is not advised for males who are still in the active period [17]. The sole approved medication for the minimally invasive therapy of PD is collagenase from Clostridium histolyticum (CCH), but in the current state of the disorder, CCH therapy is not recommended for the acute stage [18].

It is challenging to offer definitive treatment recommendations because of the large number of PD treatments that have been suggested, as well as the fact that studies on conservative treatments frequently yield inconsistent findings. Treatment for early-onset PD is particularly problematic due to the ambiguous impact of conservative medicine that follows the natural process of PD [19,20].

Despite the fact that the study protocols differed greatly, involving 6–12 injections, four trials found that taking 10 or 15 mg of intralesional verapamil significantly improved penile curvature [21–24].

Heidari et al. [23] demonstrated continued development of erectile function following an intralesional injection of 10 mg verapamil. The utilization of multiple therapeutic philosophies has resulted in significant variation in the data that are now available about intralesional therapy with verapamil. Furthermore, the results are less trustworthy because there were no placebo groups, which limits their potential to offer solid proof.

Some authors concentrated on HA as it is made up of N-acetylglucosamine and glucuronic acid, which are joined by beta-glycosidic linkages. High amounts of HA are found in the tunica albuginea, and by its hydrating function, HA controls how nutrients are distributed in connective tissue [25]. According to some studies, HA prevents interleukin (IL)-1 from inhibiting collagen formation in cultured human chondrocytes, suggesting a potential involvement in the treatment of PD [26].

In this study, we compared usage of intralesional HA and injections of verapamil in individuals with PD that were in the acute phase. Since there was no injection site ecchymosis or hematomas and no adverse medication reactions were noted, we deduced that intralesional HA had an outstanding safety profile and that patient compliance was at its highest. In comparison to intralesional verapamil, HA injection treatment was linked with a better result in pain decrease at the 12-week follow-up.

Gennaro et al. [27] study demonstrated that penile curvature and plaque size decreased in 83 individuals receiving intralesional therapy with HA compared to 81 individuals in the control group. Similar to Zucchi et al. results [28]; intralesional HA decreased plaque size and penile curvature according to a pilot research involving 65 patients. According to Favilla et al. [24], compared to intralesional verapamil, HA has a higher efficacy in relation to penile curvature and patients’ satisfaction in their prospective, double-arm, randomised, double-blinded trial. Also, Cocci et al. [14] noted that patients treated with HA had superior results than those treated with intralesional verapamil in relation to pain decrease and decrease in penile curvature.

Moreover, Au et al. [29] revealed that TNF- and IL-1-cyclooxygenase-2, inducible nitric oxide synthase, and TNF gene expression are all suppressed by avocado and soya unsaponifiables extracts in cultured chondrocytes in an LPS-stimulated monocyte/macrophage-like cell type. These pharmacological effects may be the cause of our patients’ early-onset PD patients’ decreased pain and increased satisfaction.

Additionally, it is thought that soy and avocado unsaponifiable extracts might enhance how intralesional HA affects the size of the plaque and the penile curvature [13]. Soya and avocado unsaponifiable extracts are natural compounds obtaining HA, which reduce the plaque formation and penile curvature in PD. Cai et al. [13] published a prospective randomized phase III study with 81 PD patients enrolled at two centers and randomized to oral HA administration (a combination of extracts of HA, avocado, and soy unsaponifiable substances) combined with intralesional HA (0.8% highly purified sodium salt HA 16 mg/^2^ ml) as compared with intralesional treatment only. They showed that the combination of oral and intralesional HA promoted better results in terms of curvature modifications and overall sexual satisfaction.

Additionally, the administration of intralesional HA demonstrated excellent patient compliance and no notable drug-related side effects. As a result, intralesional HA injections may be a dependable therapeutic choice for the treatment of PD’s acute phase [30].

The absence of a control group receiving a placebo and the short follow-up, which may have hindered data interpretation, are the main drawbacks of the current series. Additionally, the absence of randomization may have increased statistical bias, reducing the validity of the results. Despite these drawbacks, the results of the present series offer a solid basis for evaluating the efficiency and safety of various intralesional therapy during the acute phase of PD.

The results of the present series will need to be confirmed by other prospective, randomised, placebo-controlled studies of greater cohort sizes and extended follow-up in order to determine the true impact of intralesional HA as a treatment choice for PD in its early stages.

## Conclusion

HA is emerging as a valid choice for the treatment of PD in terms of resolution of the acute phase of the disease, and it is plausible to posit that the use of HA may contribute to the stabilization of the disease and decrease the need for the subsequent choice of a possible surgical strategy. In the acute phase, HA injections appear to be a viable minimally invasive therapy with the ability to reduce penile pain and have a stronger impact on penile curvature and patient satisfaction. Patients who received HA experienced better results and in this group of patients, a decline in penile curvature was also noted. Hyaluronic acid seems to be a better choice for intralesional therapy since it carries a reduced risk of negative side effects than other compounds.
